# Polyetheretherketone (PEEK) Into the Future: Lowering Infection Rates in Cranioplasty

**DOI:** 10.7759/cureus.72060

**Published:** 2024-10-21

**Authors:** Evan B Hughes, John Alfarone, Evan S Chernov, Nadia A Debick, Muhammad Jalal, Yeonsoo Kim, Amar Suryadevara, Satish Krishnamurthy

**Affiliations:** 1 Department of Otolaryngology, Head and Neck Surgery, State University of New York Upstate Medical University, Syracuse, USA; 2 Department of Neurosurgery, State University of New York Upstate Medical University, Syracuse, USA

**Keywords:** cranial vault deformity, cranioplasty, implant, surgery, surgical site infection

## Abstract

Introduction: Surgical site infection (SSI) is one of the most common complications following cranioplasty. At our institution, 3D printing has emerged as a popular option for creating customized polyetheretherketone (PEEK) cranioplasty implants that are lower profile than older, non-3D-printed implants. The 3D-printed implants can be integrated, with fixation plates already attached, or nonintegrated, with separate fixation plates available. To our knowledge, no study has analyzed the differences in infection rates between integrated and nonintegrated 3D-printed implants.

Objectives: This study aimed to explore whether integrated 3D-printed cranioplasty implants lead to decreased infection risk and improved patient outcomes compared to nonintegrated 3D-printed implants and further aims include exploring how operating room time and hospital length of stay affect infection risk.

Methods: A retrospective chart review of 197 patients who underwent cranioplasty surgery from 2012 to 2023 was conducted. The postcranioplasty infection rate was compared between the integrated and nonintegrated 3D implants. Pre- and postoperative antibiotic use, operating time, hospital stay length, and patient comorbidities were also evaluated. Statistical analysis was conducted using SPSS version 28.0 (IBM Corp., Armonk, NY). Initial bivariate statistics were utilized where appropriate, and ultimately, a binary logistic regression model was employed.

Results: Our overall infection rate was 12.2%, higher than the national average of 7.89%. There was no significant association between the implant type and postcranioplasty infection. However, when controlling for the clinical and demographic covariates in our study, patients who had a Charlson comorbidity index (CCI) of 3-4 were 6.36 times as likely to experience a postcranioplasty infection when compared to their counterparts with a CCI of 0, OR of 6.36, 95% CI of 1.01-40.0, and a p value of 0.048. Additionally, with every one-year increase in age, the number of infections decreased by 0.955, with an OR of 0.955, 95% CI of 0.915-0.996, and a p value of 0.031.

Conclusions: While there was no significant difference in SSI between the integrated and nonintegrated 3D-printed PEEK implants, elevated CCI and age were associated with a greater risk of infection. Our overall infection rate exceeds that reported in a recent large-scale review, which may be attributable to the higher average CCI typically observed in our tertiary care setting. Our findings illustrate that integrated and nonintegrated 3D implants offer viable and efficacious options for patients with cranial vault deformities requiring surgical repair.

## Introduction

Cranial vault deformities vary widely in terms of etiology, size, and location. The subsequent reconstructive challenge is to create an implant that is strong, malleable, and biocompatible; provides good anatomical fit and contour; and ultimately portends a low rate of surgical site infection (SSI) [1,2]. The traditionally preferred implant has been autologous bone, given its inherent biocompatibility and integrative capabilities [3-5]. That said, due to resorption or inability to maintain a sterile storage environment, bone implants are often unsuccessful, requiring further surgical management [6-8]. Alloplastic alternatives include polyetheretherketone (PEEK), polymethyl methacrylate, titanium mesh, and Norian. Advancements in 3D modeling and printing have allowed for precise fitting of the alloplastic options [9]. PEEK was created to maximize strength and minimize rigidity, thus matching the body's natural composition [1,10]. Beyond the choice of implant material, surgeons may opt to use integrated or nonintegrated implants, i.e., if the fixation plates are 3D-printed into the implant or provided separately. Historically, infection rates for alloplastic and autologous bone implants range from 5.8% to 11%, with skin flora being the common pathogen [1,5,11].

There is no clear consensus regarding which alloplastic material consistently correlates with the lowest infection rate. While a recent review by Oliver et al. compared infection rates among many of the cranioplasty implant options, there is yet to be a distinctive answer regarding the ideal treatment for these patients [5]. Some surgeons may utilize specific implant materials given personal preference, training, and comfort regardless of the outcome predictability of all available options. Given the high level of morbidity and mortality associated with SSI postcranioplasty, it is clinically relevant to determine which factors can reduce infection risk and offer a better patient prognosis. This research aim is twofold: 1) to determine if PEEK implants lead to decreased rates of SSI and 2) to explore the difference in outcomes using integrated vs. nonintegrated PEEK implants.

## Materials and methods

A retrospective chart review was conducted on 220 patients who underwent cranioplasty surgery at our institution between 2012 and 2023. The Institutional Review Board at the State University of New York Upstate (Syracuse, NY) determined that the study was exempt from IRB review with approval number 2049880-1. No patient-identifying information was included in the analysis, and informed consent was waived. The patient population comprised individuals who received either integrated or nonintegrated 3D-printed PEEK implants. The inclusion criteria included patients who required cranioplasty due to cranial vault deformities and had undergone the procedure within the study's timeframe. Exclusion criteria included patients with incomplete medical records, those who had cranioplasty performed for non-reconstructive purposes, and those who had cranial vault infections before implant placement. Only the initial procedure was included in our analysis for patients with multiple cranioplasty procedures.

Preoperative and postoperative data were collected, including demographic variables (age and sex), the Charlson comorbidity index (CCI), the presence of comorbid conditions, and details regarding the surgical procedure. The primary outcome was the occurrence of SSI, which was defined by the presence of local signs of infection (erythema, warmth, and tenderness) or confirmed by microbiological culture obtained from the wound site within 30 days postoperatively.

Additional data points included implant type (integrated or nonintegrated fixation plates), operative time (in minutes), hospital length of stay (in days), and antibiotic use (both pre- and postoperative). All patients received standard antibiotic prophylaxis preoperatively, and postoperative antibiotics were administered as needed based on individual clinical circumstances.

Statistical analysis was performed using SPSS version 28.0 (IBM Corp., Armonk, NY). Initially, bivariate analyses explored potential associations among implant type, clinical variables, and infection risk. Subsequently, multivariate binary logistic regression models were employed to adjust for potential confounding factors, including age, CCI, operative time, and hospital stay. Statistical significance was set at p <0.05.

## Results

Our analysis included 197 patients. When divided by implant type, there were 107 integrated and 90 nonintegrated implants. Most of the patients were White (81.2%) and male (68.5%). Table 1 provides a more detailed description of the patient population.

**Table 1:** Patient characteristics SD: standard deviation

Characteristic	Population (N = 197)
Sex, n (%)	
Male	135 (68.5)
Female	32 (31.5)
Age, years (mean ± SD)	42.9 ± 18.6
Race, n (%)	
White	130 (81.2)
Black	24 (12.2)
Native American	1 (0.5)
Latinx	10 (5.1)
Asian	1 (0.5)
Other	1 (0.5)
Smoking status, n (%)	
Never	80 (40.6)
Former	67 (34.0)
Current	42 (21.3)
Unknown	8 (4.1)
Indication, n (%)	
Ischemic stroke	24 (12.2)
Hemorrhagic stroke	30 (15.2)
Bleed secondary to trauma	117 (59.4)
Neoplasm or mass	12 (6.1)
Congenital	8 (4.1)
Infectious	6 (3.0)
Charlson comorbidity index, n (%)	
0	79 (40.1)
1	28 (14.2)
2	40 (20.3)
3	29 (14.7)
4	9 (4.6)
5	9 (4.6)
6	2 (1.0)
7	0 (0.0)
8	0 (0.0)
9	1 (0.5)
10	0 (0.0)
Implant type, n (%)	
Integrated	107 (54.3)
Nonintegrated	90 (45.7)
Infection, n (%)	24 (12.2)
Time between craniectomy and cranioplasty, days (mean ± SD)	157.0 ± 127.1
Length of hospital stay, days (mean ± SD)	11.5 ± 31.3
Operating room time, minutes (mean ± SD)	170.1 ± 58.0

**Figure 1:**
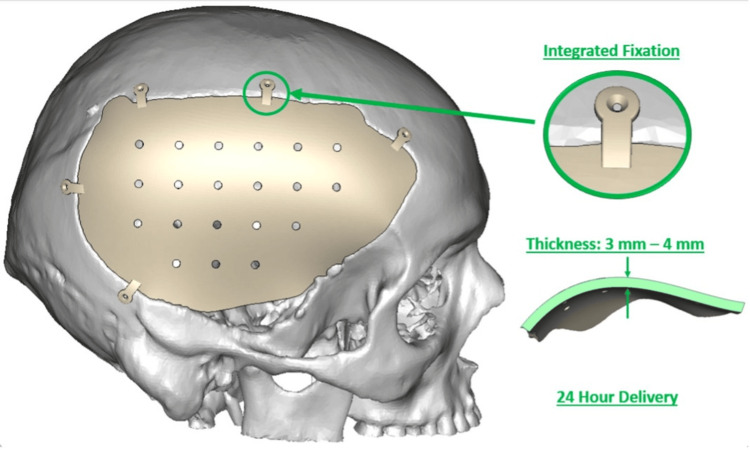
Integrated PEEK implant virtual surgical planning model PEEK: polyetheretherketone

**Figure 2:**
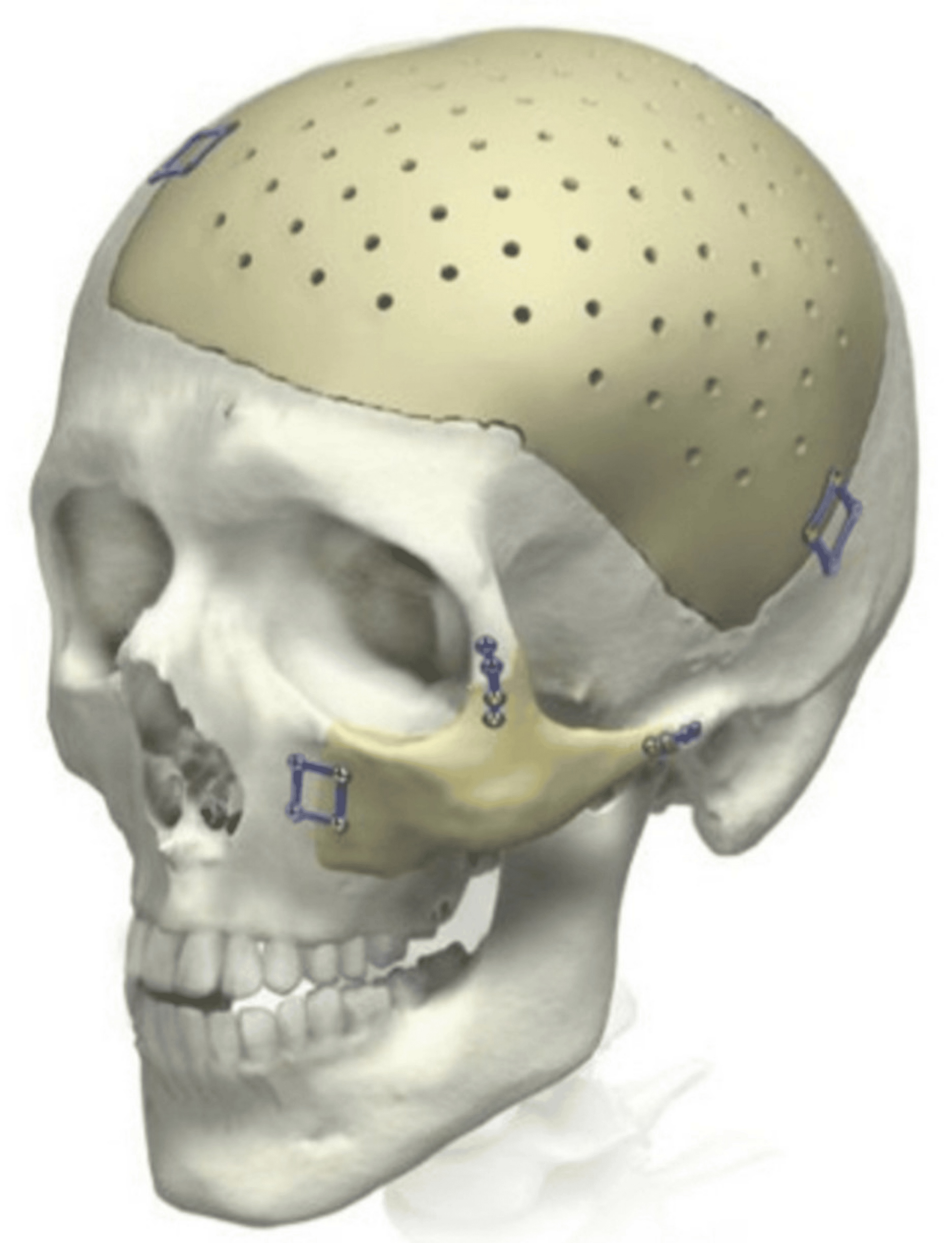
Nonintegrated PEEK implant virtual surgical planning model PEEK: polyetheretherketone

Figures 1, 2 provide a visualization of the two implant techniques.

**Figure 3:**
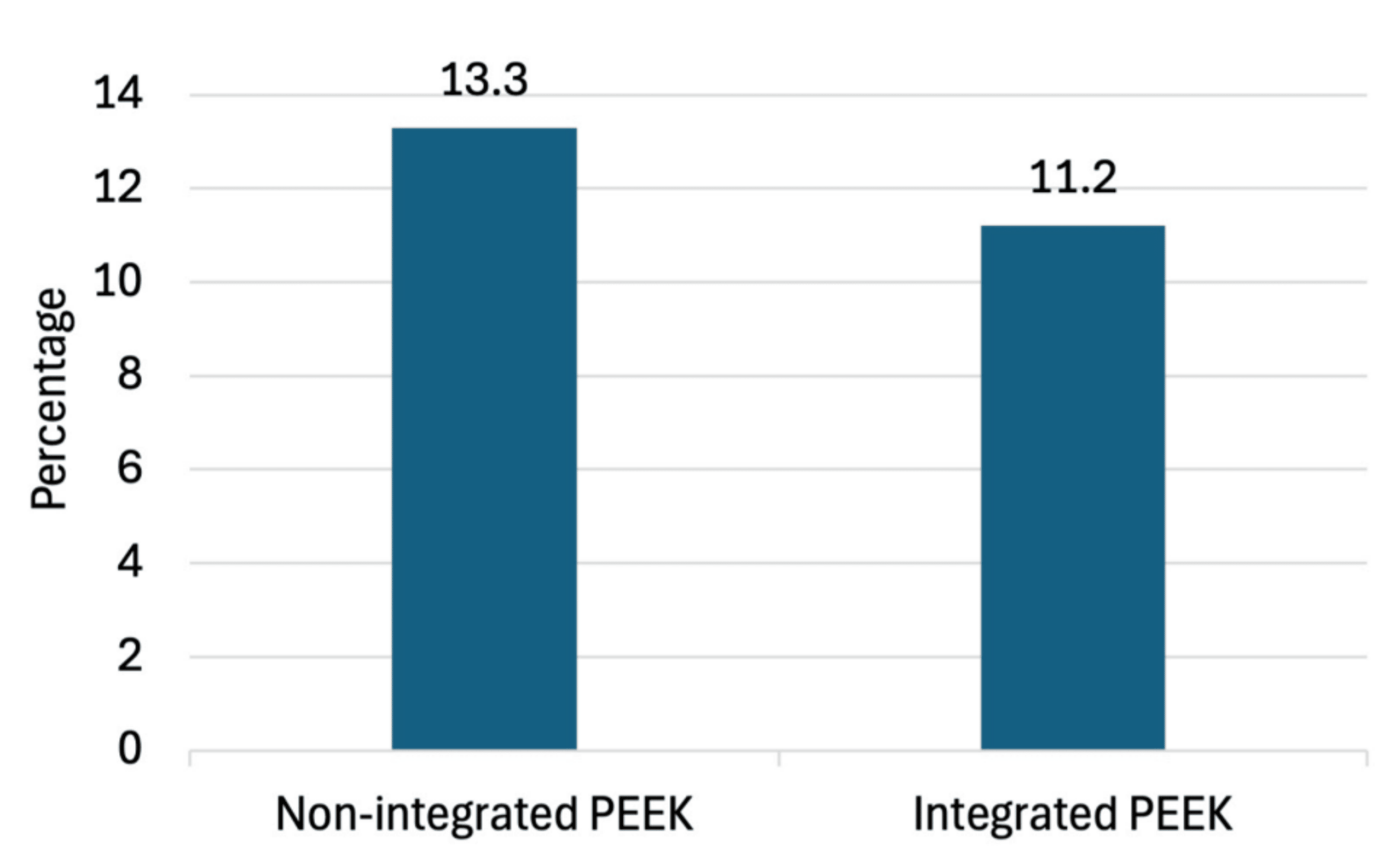
Postoperative infections by implant type PEEK: polyetheretherketone

The production of the integrated implants is locally based, thus allowing for a 24-hour delivery despite customization (Figure 1). The overall infection rate in our study was 12.2% among all patients undergoing cranioplasty (Table 1). We explored whether the implant type used during cranioplasty affected the incidence of postcranioplasty infections. The infection rates following cranioplasty with integrated and nonintegrated implants were 11.2% and 13.3%, respectively (Figure 3).

**Figure 4:**
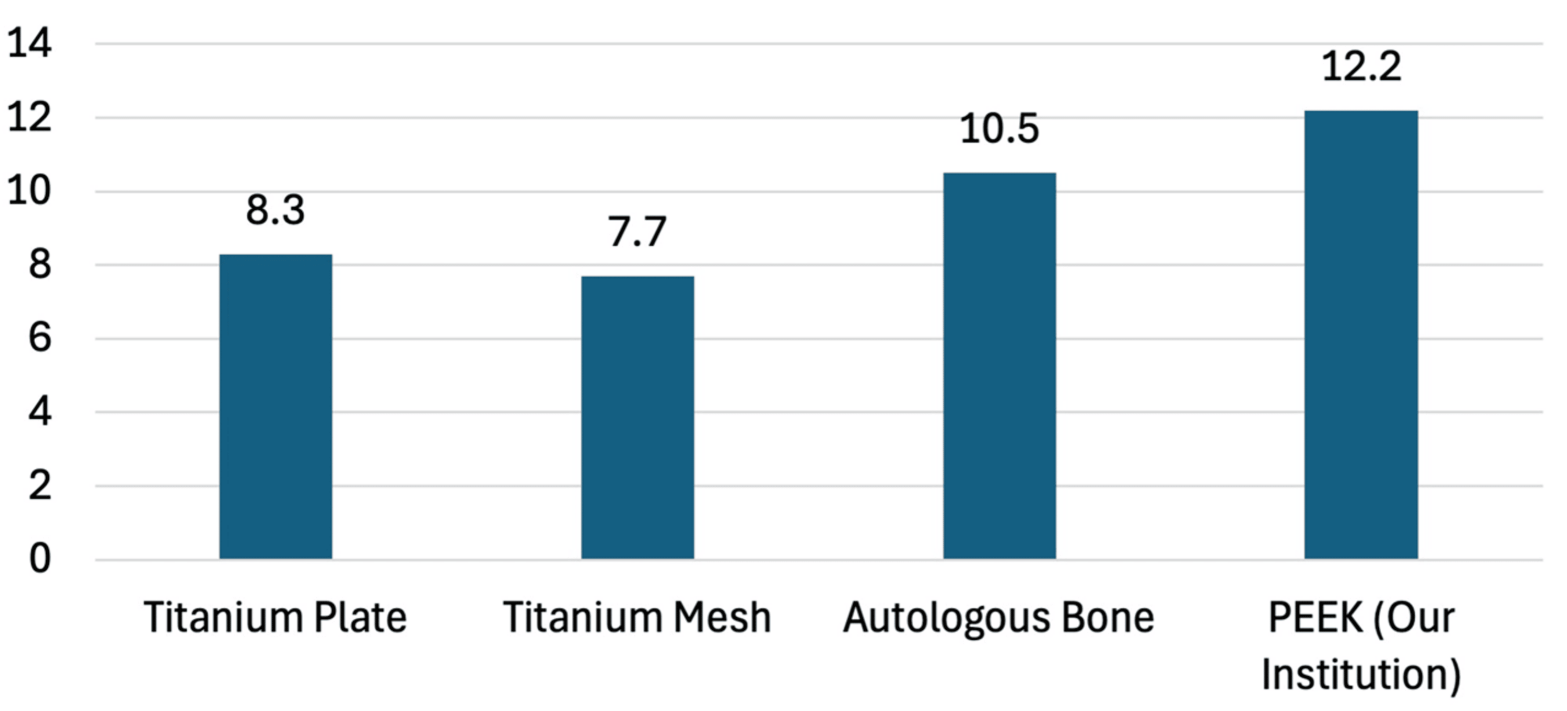
Infection rate following cranioplasty PEEK: polyetheretherketone

Bivariate analysis showed no significant association between implant type and the likelihood of infection, where X^2^(1) = 0.21 and p = 0.65. Our infection rate was higher than that of titanium plate, titanium mesh, and autologous bone, as seen in a recent systematic review (Figure 4) [1].

Further analysis was performed to investigate the clinical and demographic factors that might contribute to postcranioplasty infection risk. A multivariate logistic regression was utilized to control for these variables. One of the key findings was the impact of preexisting comorbidities on infection rates. Compared to their counterparts with a CCI of 0, those with a CCI of 3-4 were found to be 6.36 times more likely to develop a postcranioplasty infection (OR = 6.36; 95% CI = 1.01-40.0; p = 0.048).

Older patient age also significantly increased the risk for infection. Our data indicated that with every one-year increase in age, the odds of developing an infection increased slightly (OR = 0.955, 95% CI = 0.915-0.996, p = 0.031). While the effect size appears modest, the finding was statistically significant.

Several factors that we investigated did not significantly predict postoperative infection, including implant type, sex, smoking status, ventriculoperitoneal shunt placement, time in the operating room, BMI, time between craniectomy and cranioplasty, and length of hospital stay. However, female sex and current smoking appeared to confer some increased risk for postoperative infection, although not statistically significant. Further investigation with a larger and more diverse sample may help elucidate the true impact of these variables on the risk for postoperative infection.

## Discussion

Our study aimed to evaluate whether the use of integrated versus nonintegrated 3D-printed PEEK implants influences the risk of postcranioplasty SSI. While our overall infection rate of 12.2% was higher than the national average of 6.79%, we found no significant association between implant type and infection risk [5]. These findings suggest that choosing between integrated and nonintegrated fixation plates may not be a primary determinant of infection risk, contrary to our initial hypotheses. Although not significant, there was a trend toward a lower infection rate with the integrated implants.

The elevated infection rate observed in our study may be attributable to the specific patient population, many of whom presented with significant comorbidities. Our analysis revealed that patients with higher CCI scores (3-4) were over six times more likely to develop postcranioplasty infections than those with lower CCI scores. This highlights the critical role underlying health conditions play in postsurgical outcomes, particularly in complex reconstructive procedures like cranioplasty. The increased risk associated with higher CCI scores underscores the importance of careful preoperative assessment and individualized postoperative care plans for patients with multiple comorbidities. Other studies examining postoperative cranioplasty complications also found that the rate of SSI was increased in patients with higher CCI scores, specifically when the American Association of Anesthesiology score was greater than 2 [12]. It is thought that many patient comorbidities, including diabetes mellitus, autoimmune disease, and alcohol abuse, affect wound healing [11].

Age also emerged as a significant predictor of infection, with each additional year being associated with a modest but statistically significant decrease in infection risk. While this finding may seem counterintuitive given that advanced age is typically associated with worse surgical outcomes, it may reflect a selection bias toward healthier elderly patients in our cohort, or it could be due to more vigilant postoperative care in older individuals. It is also possible that younger patients requiring cranioplasties had more complex medical care that was predisposed to the need for earlier intervention. Another study investigating complications between autologous bone flaps and synthetic implants found that younger age was associated with higher rates of bone flap resorption [13]. In this same study, four out of five patients younger than 15 years of age underwent revision procedures with a synthetic implant due to osteolysis [13]. However, no definitive conclusion has yet directly connected age to infection rates for PEEK implants.

Despite the lack of a significant difference in infection rates between the integrated and nonintegrated implants, the potential benefits of each type of implant remain clinically relevant. Integrated fixation plates offer the advantage of reduced operative time and fewer foreign body components, potentially simplifying the surgical procedure. However, nonintegrated plates provide more flexibility in adjusting the fixation during surgery, which may be advantageous in cases with complex cranial vault deformities.

It is important to mention the limitations of our study. First, our patient population used Current Procedural Terminology and International Classification of Diseases codes for data extraction, which may not have fully captured all potential subjects. Second, while we attempted to adjust for known confounders in our analysis, residual confounding is possible, particularly for factors not captured in our data collection. Second, the study relied on self-reported data for variables like tobacco use, which may have introduced reporting bias. Third, while sufficient for observing most postoperative infections, the follow-up period is limited in capturing longer term trends or late-occurring events, which could lead to underestimation of certain outcomes. It is also possible that some patients presented to other healthcare centers for potential cranioplasty infection follow-up.

## Conclusions

In conclusion, this study found no significant difference in infection rates between the integrated and nonintegrated 3D-printed PEEK implants for cranioplasty. However, both patient comorbidities and age were identified as important factors influencing infection risk. As a result, individualized patient risk stratification, particularly for those with higher CCI scores, is essential in reducing the likelihood of postoperative complications. Our findings suggest that future research should focus on optimizing perioperative management strategies for high-risk patients to further improve outcomes in cranioplasty surgeries.
