# Correction: Polyetheretherketone (PEEK) Into the Future: Lowering Infection Rates in Cranioplasty

**DOI:** 10.7759/cureus.c214

**Published:** 2025-03-06

**Authors:** Evan B Hughes, John Alfarone, Evan S Chernov, Nadia A Debick, Muhammad Jalal, Yeonsoo Kim, Amar Suryadevara, Satish Krishnamurthy

**Affiliations:** 1 Department of Otolaryngology, Head and Neck Surgery, State University of New York Upstate Medical University, Syracuse, USA; 2 Department of Neurosurgery, State University of New York Upstate Medical University, Syracuse, USA

This article has been corrected as follows. The sixth paragraph of the Results section incorrectly stated that younger patient age significantly increased the risk for infection. This has been corrected to state that older patient age significantly increased the risk for infection.

